# The Shifting Epidemiology of Hepatitis A in the World Health Organization Western Pacific Region

**DOI:** 10.3390/vaccines12020204

**Published:** 2024-02-16

**Authors:** Nina G. Gloriani, Sheriah Laine M. de Paz-Silava, Robert D. Allison, Yoshihiro Takashima, Tigran Avagyan

**Affiliations:** 1 Institute of Pathology, St. Luke’s Medical Center, Quezon City 1112, Philippines; nggloriani@up.edu.ph; 2 College of Public Health, University of the Philippines Manila, Manila 1000, Philippines; 3 Accelerated Disease Control Branch, Global Immunization Division, Centers for Disease Control and Prevention, Atlanta, GA 30333, USA; rallison@americanregent.com; 4 Vaccine-Preventable Diseases and Immunization Unit, Division of Programmes for Disease Control, Western Pacific Regional Office, World Health Organization, Manila 1000, Philippines; takashimay@who.int (Y.T.); avagyant@who.int (T.A.)

**Keywords:** hepatitis A, viral hepatitis, vaccine, epidemiology, Western Pacific Region

## Abstract

Within the past few decades, improvement in sanitation and economic growth has driven a changing epidemiology of hepatitis A in the Western Pacific Region (WPR) of the World Health Organization (WHO). In this review, we gathered available published information on hepatitis A epidemiology of the countries in the WPR and reviewed the trends reported in the literature from the years 2000 to 2021. Many countries have shifted from high endemicity to low endemicity. Moreover, the administration of the hepatitis A vaccine among children in recent years has shifted disease susceptibility to the older population. Seroprevalence among children has decreased in most countries, while nearly 100% seropositivity is seen in mid adulthood. This is contrary to the epidemiology seen in previous decades when most children achieved immunity by age ten. This also presents a paradox in that better living conditions have caused more vulnerability to the older age groups who are at higher risk for severe disease. Given these trends, we recommend vaccination of vulnerable populations such as the older age groups and inclusion of the hepatitis A vaccine in government immunization programs.

## 1. Introduction

The hepatitis A virus (HAV) was first identified in 1973 and was later found to be a major cause of acute viral hepatitis in many parts of the world [1]. Acute hepatitis A is usually self-limited, though can result in fulminant liver failure and death in approximately 1% of cases [2]. The route of transmission is typically fecal–oral through person-to-person contact and contaminated food or water where conditions of poor hygiene and sanitation practices prevail, or in some cases via oral–anal sex [3].

Over the last 2–3 decades, countries have been categorized into high, intermediate, low and very low endemicity based on age of exposure and anti-HAV IgG seropositivity (immunity). High endemicity in a region is defined when ≥90% have immunity by age 10, intermediate when ≥50% have immunity by age 15, low when ≥50% have immunity by age 30, and very low when <50% have immunity by age 30 [4]. 

Changing seroepidemiology of hepatitis A has been observed in the Western Pacific Region (WPR) of the World Health Organization (WHO), with observed HAV seropositivity shifting from high to moderate to low endemicity as a result of improvements in living and socioeconomic conditions in Southeast Asia and China [5,6,7]. A more recent systematic review of the changing epidemiology of hepatitis A in the Asia Pacific countries from 1980 to 2016 showed decreasing exposure to HAV. With lower immunity to hepatitis A from childhood, older adults become more susceptible to infection [8].

Hepatitis A vaccination among children, which began in the late 1990s, also paradoxically contributed to the shift in the age of HAV susceptibility and subsequent infection towards adolescents and adults, who are much more likely to experience symptomatic and severe disease [9]. 

Hence, there seems to be a paradox in hepatitis control wherein better sanitation, higher standards of living, and childhood vaccination has led to higher risk of hepatitis outbreaks in countries transitioning from higher to lower endemicity [10]. A more systematic and comprehensive approach to reducing the burden of disease in these areas is needed. Health agencies should also explore the possibility of hepatitis A elimination in the region through targeted vaccination of specific age groups and special populations, especially since the WHO has already recommended one- or two-dose vaccination [11]. Countries must also create a strategic and systematic approach to addressing emerging outbreaks [12]. 

In this review, we searched for literature published from the year 2000 to 2022 on the seroepidemiology of hepatitis A in the WHO Western Pacific Region to review the trend of hepatitis infections in the area for the past two decades. Studies, news articles, and reports of health agencies reporting human HAV seroprevalence data in all age groups, acute viral hepatitis A outbreaks, and programs for prevention and control were included in the review. When available, information about the country, study year, study population, seroprevalence by age and other relevant information was extracted.

## 2. Vaccination Programs in Countries in the WPR

As of 2022, 5/37 (13.5%) countries or areas had introduced the hepatitis A vaccine into their routine national immunization programs in the WPR (Table 1) [13,14,15,16,17,18].

## 3. Epidemiology and Seroepidemiology of Hepatitis A in the WHO WPR

**Figure 1:**
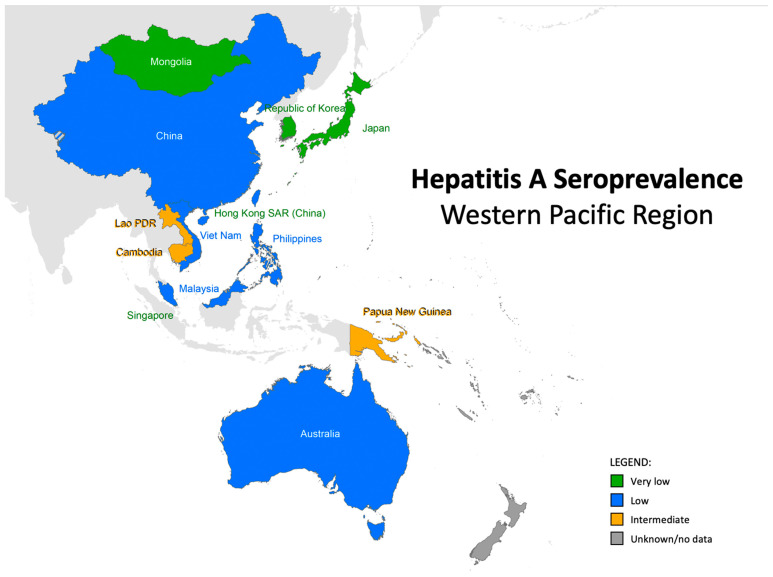
Seroprevalences of hepatitis A in the World Health Organization WPR.

The seroepidemiologic characteristics of the WHO WPR countries are shown in Figure 1. The majority of the countries have low seroprevalence. Japan and Mongolia show very low seroprevalence, while Laos, Cambodia, and Papua New Guinea have intermediate seroprevalence. 

### 3.1. Australia

Available data from Australia from 1952–1995 showed that hepatitis A notifications peaked in 1956, 1961, and 1968. After that, a gradual drop in notification rates was observed from 1971 to 1985, with small peaks noted in 1986, 1991, and 1997. From 1991–1997, notifications were reported for 15,012 cases of hepatitis A with a crude annual notification rate of 12/100,000 population. The peak notifications were in age groups 5–9 and 20–39 years. Among the case notifications, 76% were adults. The national notification rate in children was 13/100,000 per year versus 11.6/100,000 for adults per year [19].

Australia moved to a low prevalence country for hepatitis A in the 1970s to the 1990s, with a large percentage of its population rendered susceptible. Outbreaks of hepatitis A were reported to be largely from contaminated food (oysters locally grown) or from imported frozen foods in 2009, 2015–2017, 2018, and 2019, and also attributed to person-to-person fecal transmission among MSMs [20]. 

In New South Wales on 21 August 2019, at about the same time as a hepatitis A outbreak was reported in Korea, eight cases of hepatitis A were reported in Sydney and the Australian Capital Territory (ACT). The outbreak involved residents of South Korean background [21,22]. The eight cases were later linked to a food source, an imported salted or pickled clam from Koryo Food Company in Korea. 

Hepatitis A vaccination is included in the national immunization program of Australia but given only to children from indigenous populations [13]. 

### 3.2. Cambodia

A seroprevalence study in 1993 showed high HAV seropositivity in both children (27–97%) and adults (100%) [23]. Hepatitis A cases among children have been seen in hospitals and comprised 55% of admissions due to viral hepatitis [24]. In another study in 2009, among Cambodian migrant workers in Thailand, 100% of participants aged 16 years and above tested positive for anti-HAV with no age-specific trends in seropositivity [25]. The most recent nationwide seroprevalence study published in 2021 was conducted among children 5–7 years old and their mothers. They reported seropositivity of 31.5% and 91.2%, respectively. The dramatic decline in seropositivity among children suggests that there is a shift in disease epidemiology occurring, reflecting better hygiene and sanitation conditions in the country [26]. Hepatitis A is not included in Cambodia’s national immunization schedule [27].

### 3.3. China

Between 1991–1994, before hepatitis A vaccines became available, more than 70,000 cases of hepatitis A were reported per year in China [28]. However, since China introduced hepatitis A vaccine into their national immunization program in 2008, cases have significantly declined. In a study on hepatitis A surveillance and vaccine use in China from 1990–2007, hepatitis A cases declined by 90%, most dramatically among children younger than 10 years [29]. The decline in cases was attributed to improvements in socioeconomic status in addition to increasing use of hepatitis A vaccine [30].

Epidemiological characteristics of hepatitis A have changed over time. In Zhejiang Province, increased susceptibility to hepatitis A infection has been reported among adults. The mean age of hepatitis A cases shifted by more than 10 years, from 36.8 years of age in 2005 to 47.2 years in 2014 [28]. A study in Shandong province undertaken in 2019 showed significant increases in anti-HAV seropositivity, from 30.76% to 77.46% among children 1.5–7 years [31]. On the other hand, a decrease in seropositivity from 85.72% to 76.45% was seen among people of 20–29 years. This showed a positive effect of the vaccination program, but this also left the young adults susceptible. A similar shift in HAV endemicity from high to intermediate has been reported in Shijiazhuang prefecture, China [32].

A study conducted from 1990 to 2017 in Eastern, Central, and Western China with different socioeconomic conditions reported decreased hepatitis A incidence in all three regions [33]. In the Eastern region, cases decreased from 63.52 per 100,000 population in 1991–1992 to 1.18 per 100,000 in 2012–2017. Similarly, cases decreased from 50.57 per 100,000 to 1.18 per 100,000 in the Central region, and from 46.39 per 100,000 to 3.14 per 100,000 in the Western region, for the same observation periods. 

In an observational study that used China’s National Notifiable Disease Reporting System from 2004 to 2016 [34], the reported hepatitis A incidence decreased from 6.94 per 100,000 in 2004 to 1.55 per 100,000 in 2016. The highest incidence reported was in the 1–9 years age group, with incidence in the 5–9 years age group usually being higher than the 1–4 years age group. These findings were thought to be due to the impact of HAV vaccination in younger children.

In the face of a decreasing trend in hepatitis A in China since the introduction of the hepatitis A vaccine, outbreaks of hepatitis A continued to be reported as the most frequent cause of acute hepatitis. In 2006, two big outbreaks of hepatitis A were reported in southern China and northwestern China [35]. In the outbreak in the southern part of China, contaminated water was identified as the likely source, and in the northwestern part, ice cream was suspected as the source of transmission. These outbreaks were investigated using molecular epidemiologic surveillance techniques. The analysis showed genetic variability of at least 4.3% between the strains isolated from the two outbreak sites and that the prevalent strains in China were deemed to be domestic strains. Furthermore, the clustering of HAV-related isolates suggested an endemic spread. 

Between January and March 2020, an epidemic of hepatitis A was reported from coastal cities in the Liaoning province in China [36]. A 138.2% increase in hepatitis A cases in February 2020 (compared to same period in 2019) was reported and epidemiologically linked to the consumption of raw or undercooked clams, shrimp, and oysters. Adults aged 30–54 years were mostly affected (82–85% of cases) with only four cases reported in those <15 years old. Based on these findings, the authors recommended the strengthening of health education in terms of avoiding the consumption of raw or undercooked seafood, the vaccination of adults aged 20–54 years old to address population immunity gaps, and improving routine immunization and catch-up immunizations among children. Monitoring HAV genotypes in patients, the environment, and food were considered valuable in providing data required for updating prevention and control strategies for hepatitis A. 

China has a locally produced one-dose live attenuated hepatitis A vaccine and a two-dose inactivated hepatitis A vaccine that have shown >95% immunogenicity [37,38,39,40]. In the expanded program for immunization, children may be given a live HAV vaccine given as one dose at 18 months of life or an inactivated vaccine given as two doses at 18 months and 2 years of age [41]. Before 2008, the hepatitis A vaccine was categorized by China as a category 2 vaccine, and one that must be purchased and not routinely given for childhood immunization [29]. Currently, HAV live vaccines has been officially classified as category 1 (provided by the government and given free of charge) [42].

### 3.4. Hong Kong

Hong Kong in the mid-1990s and early 2000s reported that less than 20% of children and young adults had anti-HAV [43,44,45]. These rates were lower compared to the late 1980s, when around 50% of young adults were positive for anti-HAV, and, therefore, 50% were susceptible to HAV infection [46,47]. 

More detailed data showed declining anti-HAV seropositivity from 1978 to 1999. From around 44–45% positivity in 1978, it declined to 17.1% in 1987, 11.2% in 1989, and further down to 7% in 1999. This scenario increased the risk of hepatitis A outbreaks in Hong Kong from 2013 to 2017—most cases have been attributed to imported cases and person-to-person transmission among men who have sex with men (MSMs) [48,49].

In 1992, the largest outbreak happened with more than 3500 cases. In the succeeding decade, the number of cases declined and plateaued at around 500 cases per year. From 2003 onwards, the average number of cases per year continuously declined to a hundred or less. Shifting epidemiology has also been observed in Hong Kong as a majority of cases in recent years have affected patients 25–34 years of age (41% in 2016), followed by 35–44 (19%) and 45–54 (16%) age groups [50]. In an earlier report in 2006, a rightward shift of HAV age-specific seroprevalence from 1978 to 2001 was noted. In 1978, 75% of adults 21–30 had anti-HAV, while only 26.8% of individuals in the same age group had them in 2001 [51]. In a 1999 seroepidemiologic study among 1580 Chinese adolescents, the overall prevalence rate was only 7%, which was dramatically lower compared to 44.8% in 1978 and 11.2% in 1989 [45]. The shifting trend has been observed since the 1970s and 1980s [52].

The hepatitis A vaccine is not included in Hong Kong’s Childhood Immunization Program but is available in private clinics [53].

### 3.5. Japan

In Japan, hepatitis A infection is a category IV infectious disease notifiable under the Infectious Disease Control Law, amended in November 2003 [54]. An average of 266 cases of Hepatitis A per year have been reported since 2000. Most are domestically acquired. Weekly reports show a relatively low number of cases with intermittent spikes in incidence. In 2010, 2014, and 2018, a significantly higher number of cases were seen compared to the usual baseline, showing a cyclical pattern of vulnerability to hepatitis A outbreaks [54]. 

The most recent large-scale seroprevalence study in Japan was conducted in 2003, which showed overall low seropositivity for anti-HAV at 12.2% [55]. Among individuals older than 50 years old, the seropositivity gradually decreased from 96.9% in 1973 and 1984, to 74.3% in 1994, and to 50.3% in 2003. Anti-HAV seropositivity was rare in the 0–44 years age group, with a rightward shift in anti-HAV seroprevalence from 1973 to 2003 towards older age groups [50]. From this, we see a growing population of advanced age Japanese susceptible to hepatitis A infection. This is of particular concern since HAV infection in the elderly or older adults tends to be more serious [56]. In 2019, Yamamoto et al. investigated the status of HAV infection among the general population in Hiroshima using 1200 archived serum samples from Japanese residents and employees’ yearly check-ups from 2013–2015. The overall anti-HAV seropositivity for that period was 16.8%, with the 20–59 years old age group observed to be as low as 0–2%. For those aged 70 years and above, seropositivity was at 70–71%. The large number of HAV-susceptible Japanese raises the potential risk of hepatitis A outbreaks [57]. 

An outbreak in Nagano and Miyazaki prefecture occurred in 2017. Molecular analyses of patient stool specimens were conducted by the National Institute of Infectious Diseases. The results showed that epidemic strain (Genotype IA) from such patients was the same strain detected in imported frozen clams [54].

Hepatitis A vaccine is available in Japan but is not included in their National Immunization Program [58]. The vaccine can be taken voluntarily through a two-dose regimen [59].

### 3.6. Republic of Korea

Like many countries in the Asia Pacific region, HAV has been the leading cause of acute viral hepatitis in Korea. Hepatitis A became a notifiable disease in Korea in 2001. In 1997, hepatitis A vaccination was recommended to children over 12 months as part of the National Immunization Program [60] and the two-dose regimen is currently included as one of the government-supported vaccines [17]. Effectiveness of the vaccination program has been supported by the observation of increasing seropositivity in the general population following vaccine introduction; however, there is a low vaccination rate among the low-income groups [60].

A study conducted by Moon et al. (2016) analyzed hepatitis A case data from 2002–2013 reported to the Korea Centers for Disease Control and Prevention (KCDC) through the National Infectious Diseases Surveillance (NIDS) system, in addition to case data from the National Health Insurance Review and Assessment Service (HIRA) [61]. The cases of hepatitis A increased from 2005 to 2009 but decreased from 2010 to 2013. Data from the HIRA were much higher than the data from the KCDC sentinel surveillance system but with similar increasing, peaking, and decreasing patterns. Almost all of the reported hepatitis A cases between 2011–2013 were domestically acquired, and less than 0.1% of the patients were infected outside of Korea. The incidence rate was highest in the 20–39 years age group and among males.

Another seroprevalence and acute hepatitis A burden of disease study conducted in Korea from 2010–2014 showed low seropositivity in young adults [62]. Some 11,177 individuals were tested for anti-HAV IgG and were observed to have increasing seropositivity with increasing age. The 20–24 years age group showed a low 12.7% seropositivity, followed by 25–29 years at 16%, and the 30–34 years age group with 26.7% seropositivity. Moderate HAV seropositivity of 50.5% in the age group 35–39 years and 76% in the age group 40–44 years were recorded, whereas seropositivity was >90% in those 45 years of age and above. From 2009 to 2013, there was a remarkable decreasing incidence. In the 20–39 age group, incidence dropped from almost 300 cases per 100,000 population to less than 50 after 4 years. Monthly reports also showed the decline over time, with the highest peak of more than 8000 cases in May 2009, and down to less than 1000 in 2013.

In a more extensive review of the status of hepatitis A in Korea, Yoon et al. (2017) showed three decades of trends of seropositive rates by ages and years from 1979 to 2013 [63]. Seropositive rates in 2011–2013 showed a rightward shift of approximately 20 years when compared with 1979–1981 rates. These low seropositivity rates observed in adolescents and young adults in 2011–2015 could explain the rebound increase in the number of acute viral hepatitis A cases.

A hepatitis A outbreak was documented in February 2021 in a facility for the disabled where nine individuals were affected. A subsequent seroprevalence analysis was carried out among the residents in the community. There was 0% seroprevalence among those below 40 years of age, while 58.8% seropositivity was seen in the older (>40 years) age group [64]. Another hepatitis A outbreak occurred in August of 2019, where consumption of salted clams was identified as the main risk factor. Genotyping tests among the cases showed a predominance of HAV Type IA [65]. 

Overall, the decreasing HAV seroprevalence and hepatitis A incidence in Korea from 2009 to 2013 placed the population at higher risk for outbreaks. This probably explains why there have been several outbreaks since 2009, with increasing seroprevalence among the 0–10, 10–19, and 20–29 age groups from 2009 to 2019 [66]. Vaccination in the young adult age group must also be considered as they have become a vulnerable group in the country [67]. 

### 3.7. Lao PDR

Data available from Lao-PDR in 2001–2004 were from studies that looked into the infective causes of hepatitis and jaundice in 392 patients in the capital Vientiane. Some 136 patients (35.8%) 40 years and older were IgM positive. There were only 8 PCR positive patients in this cohort from whom HAV genotype 1A was isolated [68]. 

A recently published study investigated the HAV seroepidemiology in a rural province (Xiengkhouang) and the capital Vientiane. The overall anti-HAV positivity rates were 65% and 45.5%, respectively. Higher seroprevalence (35.7–62.4%, Xiengkhouang, and 11.5–69.7%, Vientiane) was seen in the 15–20 and 21–30 age groups, with almost all individuals in the older population testing positive. The lower prevalence in the urban areas was attributed to better sanitation and living conditions since the 1990s [69]. Hepatitis A is not included in their national immunization program [70].

### 3.8. Malaysia

Viral hepatitis is considered to be a major public health problem in Malaysia, primarily due to hepatitis A, B, and C. In 2000, 4067 cases of viral hepatitis were reported, most of which were hepatitis B with 2863 cases, followed by hepatitis C with 550 cases, and hepatitis A with 497 cases [71]. 

A steady drop in hepatitis A incidence has been seen over the last twenty years. The decrease has been largely attributed to government prevention and control programs introduced when hepatitis A became a notifiable disease in Malaysia in 1988. The national incidence rate for hepatitis A dropped from 2.24/100,000 population in 2000 to 0.41/100,000 in 2013. HAV was considered the main cause of symptomatic clinical cases in Peninsular Malaysia in 1996, accounting for 66.4% of the cases of viral hepatitis. This incidence dropped to 12.2.% in 2000 [71]. 

Ton et al. (1983) reported a 78.2% seroprevalence of hepatitis A in Kuala Lumpur among 110 healthy adults 18 years and older [72]. In 1985, 100% of Malaysians were reported to be seropositive for anti-HAV by 30 years of age. But in 1992, only 45% of the same age group (30 years old and above) were found to be positive for anti-HAV. The shifting epidemiology was ascribed to improvements in socioeconomic conditions and standards of living. The presence of a relatively large number of non-immune individuals opens up the population to hepatitis A outbreaks in the community [68]. 

There is particular concern among patients with chronic liver diseases (CLD), both cirrhotic and non-cirrhotic, who are at increased risk for developing serious consequences of acute hepatitis A infection. A study in 2011 was conducted which determined the seroprevalence of anti-HAV in 119 CLD patients aged 21 to >60 years of age, in Kelantan, Malaysia [73]. In this group of patients, the overall seropositivity was 88.2%, with 66.7% positivity for age group 21–30 years, and 91.3%–95.5% among the 31–60 years age group. The results showed that most of these patients with CLD greater than 30 years old already had high natural immunity to hepatitis. Routine immunization of CLD patients with hepatitis A vaccine was therefore not seen as necessary for this particular age group. Pre-screening for anti-HAV could be a cost-effective measure before offering hepatitis A vaccines for this cohort of patients.

From April to October 2002, a hepatitis A outbreak of 51 cases was reported in the district of Hulu Langat, where the source was identified to be river water with fecal contamination. A bigger outbreak affecting 800 people in Terengganu was reported in 2011 and associated with contaminated rainwater [71].

From September to October 2012, an outbreak of hepatitis A was reported in the district of Manjung, in Perak, Malaysia. The outbreak involved 78 confirmed cases of IgM anti-HAV positive individuals, most of whom were of Indian descent. Consumption of toddy, an alcoholic drink made from the sap of coconut, was linked to the outbreak, based on epidemiologic and environmental studies undertaken in the toddy processing plants and other sources [74].

Hepatitis A vaccination is not routinely given in Malaysia’s national immunization program but may be received voluntarily as per physician’s advice [75,76].

### 3.9. Mongolia

An epidemiologic study investigated anti-HAV prevalence in children aged 7–12 years from serum samples collected in 2004. Of the 520 children, 438 (84.2%) had anti-HAV [77]. In 2004, another study looked at the anti-HAV prevalence among adults aged 23–86 years old and found that 100% were positive for anti-HAV IgG [78]. 

A review of records from January 2012 to December 2014 in Ulaanbaatar Hospital, Mongolia was conducted to examine cases of acute viral hepatitis. Of the 278 cases of hepatitis A, 41%, 44%, and 14% were found in the 2–9, 10–19, and 20–29 age groups, respectively. There were no patients 40 years old and above [79]. 

Mongolia was highly endemic of hepatitis A until the introduction of hepatitis A vaccination in 2012. Although not part of the expanded program on immunization, the vaccine is fully funded through their domestic immunization fund. The classic seasonal spikes in hepatitis A cases were seen before 2012, but the number of cases started to decline without the expected seasonal peaking in 2013 after the vaccine was introduced. Mongolia successfully achieved 95.9% coverage of the two-dose vaccine given at 14 months and 2 years of age [80]. 

### 3.10. New Zealand

Very limited current data are available for New Zealand, since the incidence of hepatitis A was observed to have decreased sharply since the 1960s. About half of recently reported cases had a history of overseas travel [81]. In 2016, the Environmental Science and Research Institute (ESRI) of New Zealand reported an outbreak of 7 cases of hepatitis A associated with consumption of frozen strawberries and blackberries from China, with a 71% hospitalization rate [82]. Hepatitis A vaccine is not included in their national immunization schedule but is government funded for high-risk groups [83].

### 3.11. Papua New Guinea

Seroprevalence data for Papua New Guinea (PNG) is scarce. The only data available were from a 1986 seroprevalence study among expatriates in PNG [80]. The study was conducted among 380 missionaries and their dependents who showed 47% anti-HAV positivity, increasing the longer they stayed in PNG. Around 50% of those below 21 years old were anti-HAV IgG positive. It was concluded that these missionaries and their children who were seronegative when they started to live in PNG could be considered a particular risk group who could benefit from hepatitis A immunization [84]. The national immunization program of PNG does not include the hepatitis A vaccine [85]. 

### 3.12. Pacific Island Countries and Areas

There are limited published data on the epidemiology of hepatitis A in the Pacific islands and the status of hepatitis A vaccination in the area. What makes the region interesting is their relative geographic isolation from their neighbors which raises questions on whether they have similar trends in hepatitis A epidemiology as in other countries. 

Some of the earliest reports date back in 1970s. The overall seroprevalence in 1979 in selected islands in Micronesia, Melanesia, and Polynesia was about 90% by age 15–19 [86]. In Fiji (Viti Levu), Tuvalu (Funafuti), Niue, Cook Islands (Ratonga), and Samoa (Upolu), high overall seroprevalence was also seen at 84.3%, 79.9%, 95.2%. 95.0%, and 81.6%, respectively [87]. Most infections seemed to occur in the first decade of life and peaked in the second decade. There was increasing or sustained prevalence at older age groups. In many of these islands, seroprevalence among children less than 10 years old was at least 20% and as high as 50–100%, with only a few exceptions [86,87]. 

In more recent years, the seroprevalence was much lower in children in the first decade of life. In a study investigating hepatitis A from 1995–2008, the seroprevalence in Micronesia (Chuuk and Pohnpei States) was less than 10% in children less than 10 years old. In Samoa, there were no seropositive among children less than 6 years old. Only the Marshall Islands had high seroprevalence of around 80–90% in children in the first decade of life which indicates higher endemicity in the area [88]. 

Pohnpei State in Micronesia is interesting among the islands in that it showed a very dynamic trend in hepatitis A seropositivity. In 1963, there was zero seroprevalence until age 19 with a dramatic rise of up to 75% among individuals age 20–29. Ninety percent prevalence was only observed in individuals 30–39 years old. After an outbreak in 1973, repeat testing showed that all individuals aged 14–21 were seropositive for hepatitis A [86]. In a 1995–2008 report, 0.8% of children were seropositive, while 95% of adolescents tested had anti-HAV [88]. 

There had been no outbreaks in Pohnpei since the 1970s until one emerged in 2008 with adolescents representing a majority of the patients. The outbreak was attributed to two travelers from Chuuk State which was experiencing an ongoing outbreak during that time [89]. Pohnpei, therefore, seemed to have very little exposure to the virus in the earlier decades, but greater mobility in the more recent years could have introduced and reintroduced the exposure to the population. Because infants and children are not usually exposed, it is not surprising that adolescents and young adults become the more susceptible population when another outbreak emerges. 

A more recent outbreak was described in 2016–2017 in the Marshall Islands where the median age of patients was 8 years old [90]. 

Although the beginnings of a shift in the epidemiology is also seen in the Pacific Islands, the shift is not as great as in Japan and Korea. We can expect, however, that with this trend, the nature of future outbreaks may evolve closer to these bigger countries, especially with continuous socioeconomic growth and mobility. 

### 3.13. Philippines

Seroprevalence studies conducted in Metro Manila among middle income communities in 1993 showed an overall seropositivity of 62.3% with anti-HAV positivity increasing with age, from 18.4% in children less than 10 years of age, to 95.8% in those 61 years and above [91]. In an unpublished study in the Philippine Health Research Registry, the overall seropositivity in 2021 is relatively lower at 41.5% in urban areas and 44.2% in rural areas [92]. Their findings suggest that the endemicity is considered low. This shift in epidemiology mirrored the downward trend also occurring in the Southeast Asian, Western Pacific, and Asia Pacific regions [5]. 

Hepatitis A is a notifiable disease in the Philippines [93]. From 2015 to 2019 hepatitis A cases were reported in almost all 17 regions of the country, with cases tending to decrease yearly. There were 839 confirmed cases notified in 2015, 666 cases in 2016, 422 cases in 2017, 76 cases in 2018 and 152 cases during the first 7 months of 2019. Some regions reported increases in the number of cases from 25% up to 1000% [94,95,96,97,98]. 

Five hepatitis A outbreaks were reported which were verified by the Event-based Surveillance and Response unit (ESR) of the Department of Health. These occurred in 2010 in Iloilo Province, in 2012 in the Cordillera Autonomous Region (CAR), and in 2014, 2015, and 2016 when hepatitis cases were verified by ESR but no details could be accessed [88,89,90,91,92]. Two outbreaks of hepatitis A in Hawaii and at least 3 or 4 cities in the USA were also reported and linked to consumption of HAV-contaminated scallops and yellow fin tuna from imported products from the Philippines [99,100]. 

Hepatitis A vaccine is not included in the country’s National Immunization Program. 

### 3.14. Singapore

The last 2–3 decades saw tremendous socio-economic progress in Singapore, with concomitant improvement in standards of hygiene, sanitation and living conditions. An epidemiologic study from 1990 to 2009 showed that the indigenous cases of hepatitis A declined from 1.8/100,000 in 1989 to 0.7/100,000 in 2009. The overall hepatitis A prevalence declined from 31.8% in 1984–1985 to 25.9% in 1993 [101]. This changing prevalence in hepatitis A infection made a large segment of its population vulnerable to the disease, especially in view of the high volume of travel into and out of the country, as well as its domestic consumption of imported foods such as shellfish. 

More recent incidence data from the Communicable Diseases Surveillance Report classified under Food-borne diseases, yielded 81 notifications of hepatitis A in 2017 with an incidence rate of 1.4/100,000 population, compared to 48 cases in 2016 [102]. Of the 81 cases, more than half were indigenous cases, and 32 were imported cases, mostly from India (7 cases) and Southeast Asian countries: Malaysia (4 cases); Indonesia and Thailand (3 cases each); 2 cases each from Myanmar, Philippines, Cambodia, Vietnam, Taiwan and Australia; and 1 case each from Sri-Lanka, Hongkong SAR, South Korea and Uzbekistan. From 2013 to 2016, more notifications received for hepatitis A were imported cases, whereas in 2017, there were more local cases than imported ones. Singapore’s importation of seashells, like cockles and oysters from coastal areas in countries in the region, were associated with massive hepatitis A outbreaks in the 1970s to 1980s [101]. 

The HAV seroprevalence in Singapore shows very low-level transmission similar to developed countries like Japan, Australia, and New Zealand. However, its location within a region highly or moderately endemic for HAV infection, and the high mobility within the region makes it highly vulnerable to introduction of hepatitis A. Hepatitis A is not included in Singapore’s national childhood immunization schedule [103]. 

### 3.15. Vietnam

Similar to other low-to middle income countries in the WHO Western Pacific Region, data available for HAV seroprevalence in the Mekong River Delta region of Vietnam are rare. One available publication in 1999 showed increasing anti-HAV seropositivity with age where children 5–9 years old already reached 95% seropositivity [104].

Reports from Vietnam show an apparent epidemiologic shift as well. In a 1981 study that recruited 564 Vietnamese refugees, 90% were positive for anti-HAV at 15 years old [105]. However, in a recent study that looked at 422 adult female immigrants from Vietnam, only 59% were seropositive [106]. Genotypic characterization of the virus showed that genotype 1A is the most common among pediatric patients diagnosed with hepatitis A [107]. Hepatitis A is not included in the country’s National Immunization Program [108].

## 4. Discussion and Recommendations

Shifting patterns of anti-HAV positivity, with a transition from higher to lower seroprevalence, has been notable in various countries of the WHO WPR, particularly in Singapore, Republic of Korea, Hongkong (SAR), China, Malaysia, Vietnam, and the Philippines. Hepatitis A vaccination has been included in the national immunization programs of at least five WHO WPR countries (Australia, China, Republic of Korea, Mongolia, and New Zealand) [13,14,15,16,17]. Most of the other countries have available HAV vaccines but are not funded by government. 

In countries that included the HAV immunization, drastic decrease in the number of cases were observed upon inclusion of HAV vaccines in the national immunization program. China and Mongolia, for example, had previously very high numbers of cases but later transitioned to much lower endemicity in the recent years. 

One emerging problem in these countries, however, are the immunity gaps. Because children were targeted for immunization, the susceptibility to hepatitis A infection shifted to the older populations who are more vulnerable to severe disease. This includes young adults who did not have HAV vaccines as children. In both China and Korea, young adults comprised a big portion of cases during outbreaks, which may suggest that this age group had lesser exposure to the virus because of better living conditions but are unprotected because of no vaccination during childhood. A systematic review conducted by Gripenberg et al. in 2018 showed that the risk of exposure to hepatitis A continued to change over time in many of these countries, with clinical hepatitis A infections seen now more frequently in older age groups [8]. 

Better living conditions have also contributed to lesser HAV endemicity as observed in high income countries like Japan and Hongkong. Despite better sanitation, however, mobility within the region enables transmission across countries such as seen in outbreaks in Australia, Malaysia, and the Pacific Islands. Susceptible adults usually comprise a big proportion of the affected populations in outbreaks. 

While most countries in the WHO WPR have low to very low endemicity, some countries such as Cambodia, Lao PDR, and Papua New Guinea still have intermediate endemicity with most adolescents being seropositive to HAV. Interestingly, these countries also observed a trend towards lesser infections and lower seroprevalence in recent years, which reflect better living environments. More published data, however, is needed to better capture the status of HAV exposure and infections in these countries. 

The findings of this review should be interpreted considering the following limitations. First, it is difficult to have comprehensive understanding of changing epidemiology of hepatitis A in some countries of the WHO Western Pacific region due lack of seroprevalence studies and limited information on surveillance and disease trends over the years. Second, for several countries, available publications review trends in hepatitis A epidemiology more than ten years prior to this review, which limits understanding of the current situation with hepatitis A in these countries. Third, there is limited understanding of the burden of hepatitis A in intermediate endemicity countries that have not yet introduced the vaccine, like Cambodia, Lao PDR and Viet Nam. Fourth, socioeconomic, environmental, behavioral and other risk factors that contribute to the emergence of outbreaks despite better living conditions should be assessed in more details.

New strategies are thus needed to address the emerging challenges of hepatitis A control in WHO WPR. 

HAV vaccination is a crucial primary measure in hepatitis control [109]. In 2022 the WHO updated a position paper on Hepatitis A vaccine, which recommends that vaccination against hepatitis A virus be introduced into national immunization schedules for individuals aged ≥12 months, if indicated on the basis of: (i) an increasing trend over time of acute hepatitis A disease, including severe disease, among older children, adolescents or adults; (ii) changes in the endemicity from high to intermediate; and (iii) considerations of cost–effectiveness [110]. With the shift in epidemiology, however, vaccination coverage may also be extended to adults and elderly in high-risk setting and to other special populations such as men who have sex with men, and persons experiencing homelessness [111,112,113]. 

WHO also recommends that following the introduction of hepatitis A vaccines, their impact should be regularly assessed using morbidity and mortality surveillance data. Better surveillance may be carried out through molecular epidemiology and genotyping to provide insight on transmission dynamics during outbreaks and to identify viral reservoirs in the environment [114]. There is also need for increased capacity building on the availability of molecular methods for surveillance. 

Moreover, updated seroepidemiologic data are needed, especially in areas where published research is limited. One limitation, for example, that we encountered in this review is the difference in the amount of published data among the countries. Some countries, such as Japan and China, have numerous published surveillance data, while others, such as Cambodia, have limited available literature. Furthermore, since not all papers reported the sensitivity or specificity of the used serologic tests, we could hardly assess potential biases in the reported seroprevalence data. 

With increasing mobility and travel, control of HAV in one area may easily be disrupted with transmission from other endemic countries. Concerted efforts among neighboring governments are thus imperative to effectively eliminate HAV in the region. Sharing of best practices and collaborative public health programs will be needed to eliminate HAV infection in the WHO WPR. 

## Figures and Tables

**Table 1:** Countries in the WHO WPR offering hepatitis A vaccines in their national immunization program.

Country	Description	Number of Doses	Schedule	Route	Coverage
Australia	Hepatitis A inactivated vaccine (Vaqta^®^ Paediatric)	2	12 months, 18 months	IM	Aboriginal and Torres Strait Islander people in Queensland Northern Territory, Western Australia, and South Australia
China	Hepatitis A vaccine, live (HepaA-L)	1	18 months	SC	Entire country
	Hepatitis A vaccine, inactivated (HepA-I)	2	18 months, 2 years	IM	Entire country
Mongolia	Hepatitis A inactivated vaccine	2	14 months, 2 years	IM	Entire country
New Zealand	Hepatitis A inactivated vaccine (Havrix)	1	16+ years	IM	Transplant patients, close contact of hepatitis A cases
	Hepatitis A inactivated vaccine (Havrix Junior)	1	1 to 15 years old	IM	Transplant patients, children with chronic liver disease, close contacts of hepatitis A cases
Republic of Korea	Hepatitis A inactivated vaccine	2	12 to 24 months	IM	Entire country

## Data Availability

No new data were created or analyzed in this study. Data sharing is not applicable to this article.
